# Direct Mode I Fracture Toughness Testing Using Short Rod Specimens: A New Loading Fixture

**DOI:** 10.3390/ma19183926

**Published:** 2026-09-15

**Authors:** Rashid Hajivand Dastgerdi, Łukasz Ostrowski, Vahab Sarfarazi, Agnieszka A. Malinowska

**Affiliations:** 1 Faculty of Geo-Data Science, Geodesy, and Environmental Engineering, AGH University of Krakow, 30-059 Krakow, Poland; amalin@agh.edu.pl; 2 Faculty of Civil Engineering and Resource Management, AGH University of Krakow, 30-059 Krakow, Poland; lukost@agh.edu.pl; 3 Department of Mining Engineering, Hamedan University of Technology, Hamedan 65155-579, Iran; sarfarazi@hut.ac.ir

**Keywords:** Short Rod test, Mode I fracture toughness, direct shear apparatus, discrete element method (DEM)

## Abstract

Reliable determination of Mode I fracture toughness is essential for evaluating crack initiation and propagation in rock engineering applications such as hydraulic fracturing, underground energy storage, geothermal energy extraction, and CO_2_ sequestration. The Short Rod (SR) test is the only ISRM-suggested direct method for determining Mode I rock fracture toughness, but its wider application is limited by a relatively complex loading system. This study proposes a simple shear-to-tensile (ST) fixture for conducting SR tests using a conventional direct shear apparatus. The CNC-cut steel fixture converts the applied shear load into direct tensile loading on the specimen. Tests on gypsum produced symmetric fracture paths and consistent load–displacement responses, with a mean $K_{IC}$ of 0.287 ± 0.008 MPa√m. SCB tests on the same material yielded 0.288 ± 0.020 MPa√m, showing close agreement between the two methods. DEM simulation further captured fracture initiation at the chevron-notch tip and progressive crack propagation through the specimen ligament. The results demonstrate the feasibility of the proposed configuration as a proof of concept for direct SR testing, with future studies aimed at extending its validation to stronger rock materials.

## 1. Introduction

**Figure 1:**
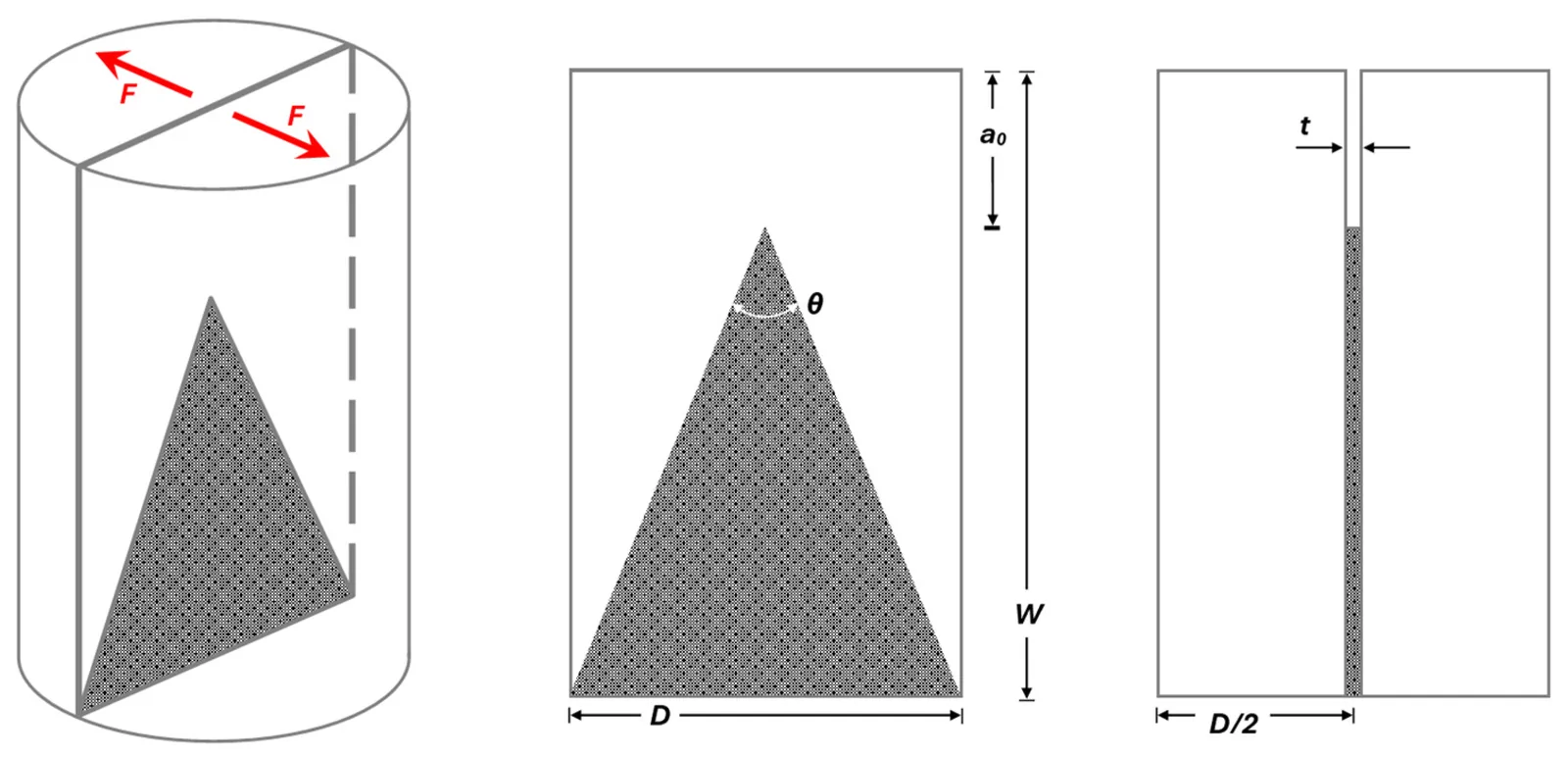
Geometry and associated notations of the ISRM suggested Short Rod specimen: W = 1.45D, a_0_ = 0.48D, θ = 54.6°, t = 0.03D or 1 mm, and D > 10 times the grain size.

Fracture toughness is a fundamental material parameter that quantifies the resistance of a material to crack propagation in the presence of preexisting flaws. In rock mechanics, this property is particularly important due to the inherent heterogeneity of rocks. The presence of mineral grains, pores, and microcracks creates local stress concentrations and promotes crack initiation and propagation, particularly in brittle rocks [1,2,3]. Accurate determination of fracture toughness is therefore essential for the analysis and design of rock engineering applications and structures, including hydraulic fracturing, underground energy storage, geothermal systems, drilling and blasting operations, tunnels, and underground excavations, where crack propagation may directly affect performance, stability, and safety [4,5,6,7,8,9]. Within the framework of linear elastic fracture mechanics (LEFM), fracture is classified into three fundamental modes: Mode I (tensile opening), Mode II (in-plane shear), and Mode III (out-of-plane shear) [10]. Correspondingly, fracture toughness is defined for each loading mode. For brittle geomaterials, Mode I fracture toughness $K_{IC}$ is generally considered the most relevant parameter because rocks exhibit low tensile strength and are highly susceptible to tensile cracking [11]. To standardize the determination of $K_{IC}$ in rocks, the International Society for Rock Mechanics (ISRM) proposed several laboratory methods, including the Short Rod (SR), Chevron Bend (CB), Semi-Circular Bend (SCB), and Cracked Chevron Notched Brazilian Disc (CCNBD) tests [12,13,14]. These methods use specific specimen geometries and loading configurations to promote Mode I crack initiation and propagation. However, many rely on indirect loading conditions such as bending or diametral compression, which may introduce additional stress components near the crack tip and influence the measured fracture toughness. Consequently, the measured $K_{IC}$ may vary among test configurations, even for the same rock, because differences in crack-tip stress state and crack-growth conditions affect the apparent fracture toughness [15]. Notch geometry also influences fracture process zone development. Chevron-notched specimens can promote stable progressive crack growth and reduce sensitivity to initial notch length and notch-tip preparation, which is advantageous for fracture toughness determination [16]. Among these methods, the Short Rod test is considered a direct method. The ISRM-recommended specimen geometry and dimensional proportions are shown in Figure 1 [17].

The SR test employs a chevron notched cylindrical specimen subjected to diametral tensile loading, which promotes stable crack initiation and controlled crack propagation. In the conventional configuration, steel end plates are adhesively bonded to both specimen ends, and a loading link system is attached to these plates. The tensile load is then applied through grips that pull the link system in opposite directions [11,17]. Many studies have proposed or improved loading fixtures to simplify test execution, enhance measurement accuracy, and enable testing under different conditions [18,19,20,21,22]. For example, the SR specimen and its associated loading fixture have been modified to extend their applicability to various quasi-brittle materials and testing conditions, including elevated temperatures, dynamic loading, and anisotropic behavior. These modifications include introducing a bottom groove for direct load application instead of bonded end plates [23,24], using mechanically fastened configurations with bolts and steel plates rather than adhesive grips [25,26], and adopting wedge based loading systems under uniaxial compression [27,28,29,30,31]. Such adaptations aim to simplify the experimental setup, improve load transfer stability, and facilitate practical implementation while preserving the fundamental fracture mechanics basis of the method. Besides the demanding specimen preparation required for the SR test, the lack of a simple and easily manufacturable loading fixture remains a key limitation for its wider use in routine laboratory practice.

Hajivand Dastgerdi et al. (2026) proposed a new loading fixture, referred to as the shear-to-tensile (ST) tool, which can be integrated with a conventional direct shear apparatus to enable direct Mode I fracture toughness testing using pseudo-Compact Tension (pCT) specimens [32]. The proposed configuration was experimentally validated using gypsum specimens as representative rock like materials. The obtained fracture toughness results were compared with those from SCB and cracked straight-through Brazilian disc (CSTBD) tests, showing close agreement, particularly with the SCB method. The average $K_{IC}$ values obtained from dry pCT, SCB, and CSTBD tests were approximately 0.178, 0.188, and 0.274 MPa√m, respectively. The study also demonstrated that the proposed setup could capture the post-peak response of the specimens. Building on this, the present study investigates the application of the ST loading fixture to Short Rod (SR) testing. For this purpose, SR tests were performed on gypsum specimens, and the obtained $K_{IC}$ values were compared with those from SCB tests. In addition, a three-dimensional DEM model was developed to examine fracture initiation and propagation within the SR specimen under the proposed loading configuration. The proposed configuration aims to provide a simple, cost-effective, and easily manufacturable alternative for SR testing while preserving the fracture mechanics basis of the conventional SR method.

## 2. Materials and Methods

### 2.1. Specimen Preparation

**Figure 2:**
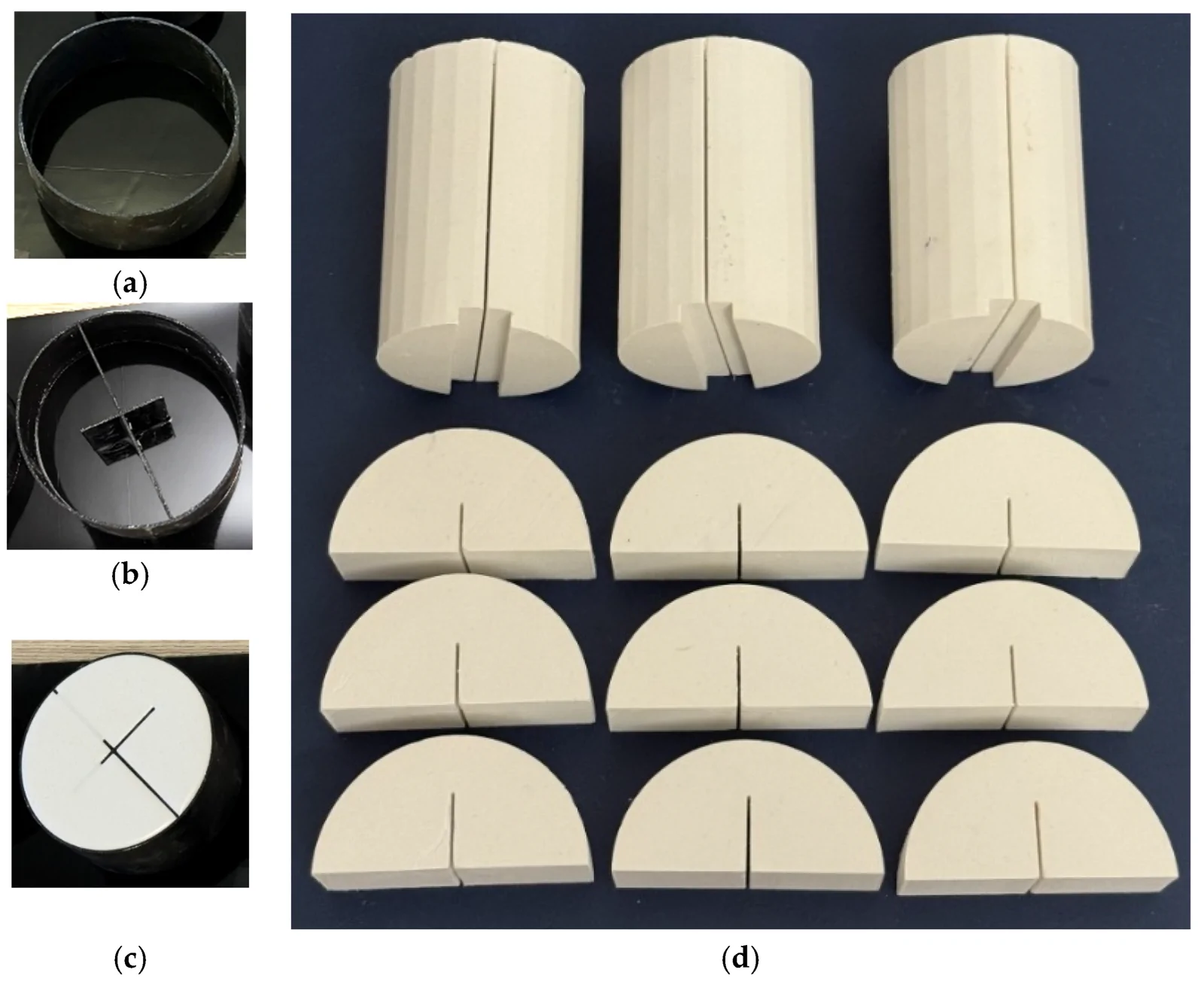
Overview of the specimen fabrication process: (**a**) mold assembly and sealing; (**b**) placement of coated inserts; (**c**) pouring of the gypsum mixture; (**d**) SR and SCB specimens after removal from the molds.

Gypsum was selected as a representative brittle geomaterial to evaluate the pro-posed loading configuration. Cast artificial materials such as gypsum and cement are commonly used in fracture studies due to their high geometric accuracy and material uniformity, which reduce the effects of natural rock heterogeneity and specimen preparation errors [32,33,34,35,36,37]. The material in this study was prepared by mixing gypsum powder and water at a mass ratio of 2:1 to obtain a uniform slurry, which was then poured into the 3D printed molds. Figure 2 illustrates the preparation procedure for the SCB specimen together with the fabricated SR and SCB samples. Flexible molds were used for the SR specimens to facilitate easy removal after casting, while jointed plastic molds were used for the SCB specimens.

After demolding, specimens were stored under laboratory ambient conditions for a curing period of six weeks. The specimen types, quantities, and geometric dimensions are summarized in Table 1. A minor modification was introduced to the SR specimens by incorporating a bottom groove at the specimen base, following previous studies [23,24,25]. The groove acts as a mechanical grip for load transfer from the ST tool and simplifies specimen preparation for cast materials such as gypsum by eliminating the need for adhesively bonded end plates. The groove extends along the specimen base with a depth of 5 mm and a width of 15 mm.

### 2.2. ST Loading Fixture

**Figure 3:**
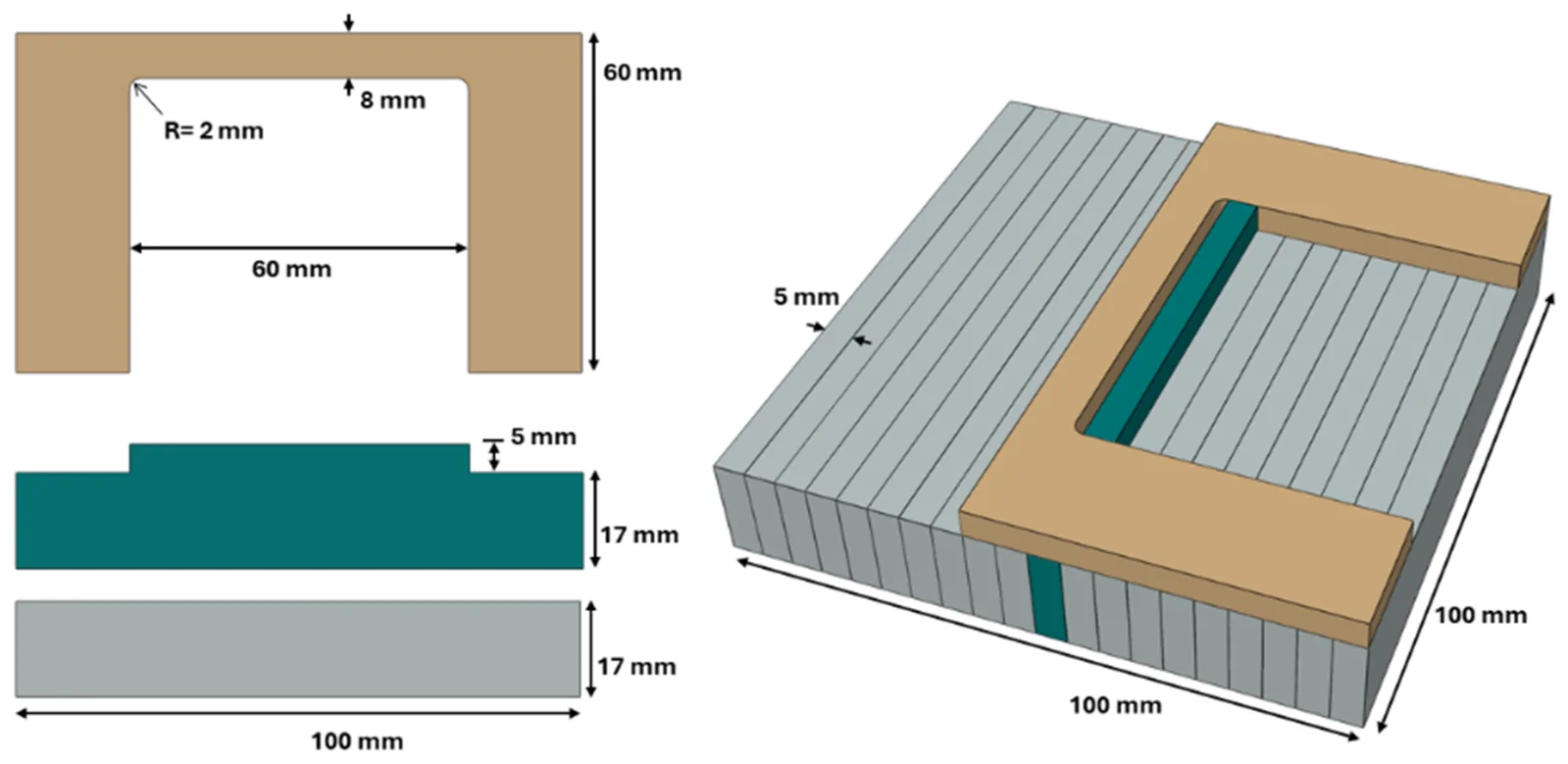
Geometry and components of the shear-to-tensile (ST) fixture.

The ST tool enables tensile loading of SR specimens using a conventional direct shear apparatus commonly available in geomechanics laboratories. The apparatus used in this study has a maximum load capacity of 8 kN and allows programmable displacement rates between 0.0001 and 15 mm/min. A square shear box with internal dimensions of 100 × 100 mm was used, which is sufficient to accommodate 60 mm diameter SR specimens. The ST tool is fabricated from 5 mm thick S235 structural steel plates using CNC cutting and can be easily installed and removed within the shear box. In this configuration, the applied shear force is converted into axial tensile loading acting on the specimen. The geometry and components of the ST fixture are shown in Figure 3. The dimensions were optimized through finite element simulations to ensure adequate stiffness and strength under loading.

**Figure 4:**
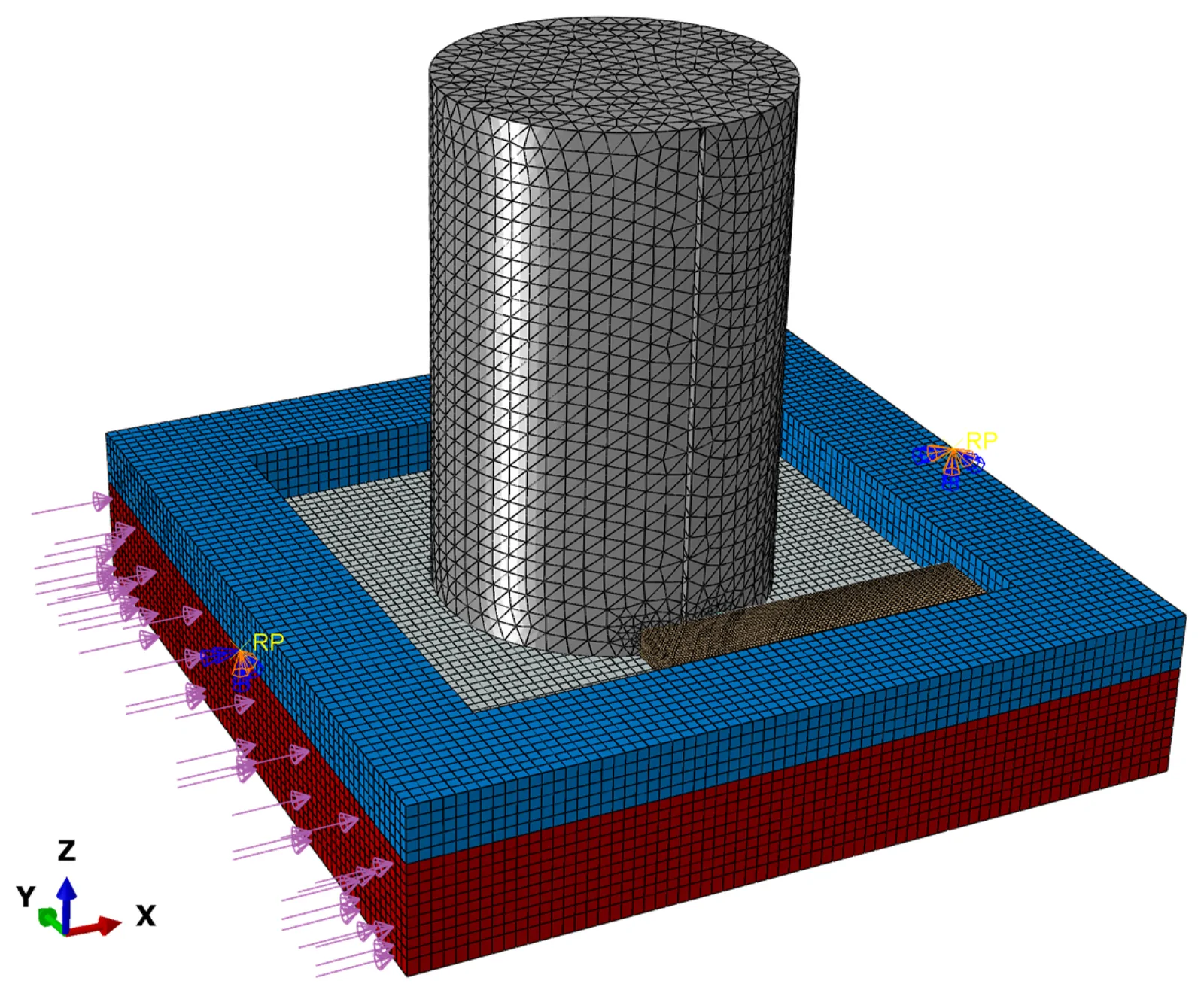
Finite element model developed for the ST fixture analysis, showing the meshed components and applied boundary conditions.

The lower assembly consists of 19 rectangular plates that fill the bottom shear box and stabilize a central plate acting as a hanger engaging the groove at one end of the SR specimen. A U-shaped plate engages the groove at the opposite end and transfers the load to the upper shear box. In this configuration, the direct shear apparatus operates in its standard mode while continuously recording load and displacement. For structural assessment of the ST tool, a three-dimensional finite element model was developed in ABAQUS (version 2024), following a similar approach to that reported in the literature [22,38,39]. All steel plates were modeled as linear elastic with an elastic modulus of 210 GPa and a Poisson ratio of 0.3. A 60 mm diameter Short Rod specimen was included to capture the plate -to-specimen interaction and was assigned representative stiff rock properties with an elastic modulus of 55 GPa and a Poisson ratio of 0.3. The upper shear box was fully restrained, while the lower box was allowed to translate only in the horizontal direction. A horizontal load of 5 kN was applied to the lower box over 60 s. The upper and lower shear boxes were modeled as rigid shells using R3D4 elements, while all steel plates were discretized with C3D8R elements. A locally refined mesh was adopted in the contact regions and in areas where stress concentrations were expected. Surface-to-surface contact was defined at all interfaces, with hard normal contact and Mohr Coulomb friction in the tangential direction. The FEM mesh and boundary conditions are shown in Figure 4.

**Figure 5:**
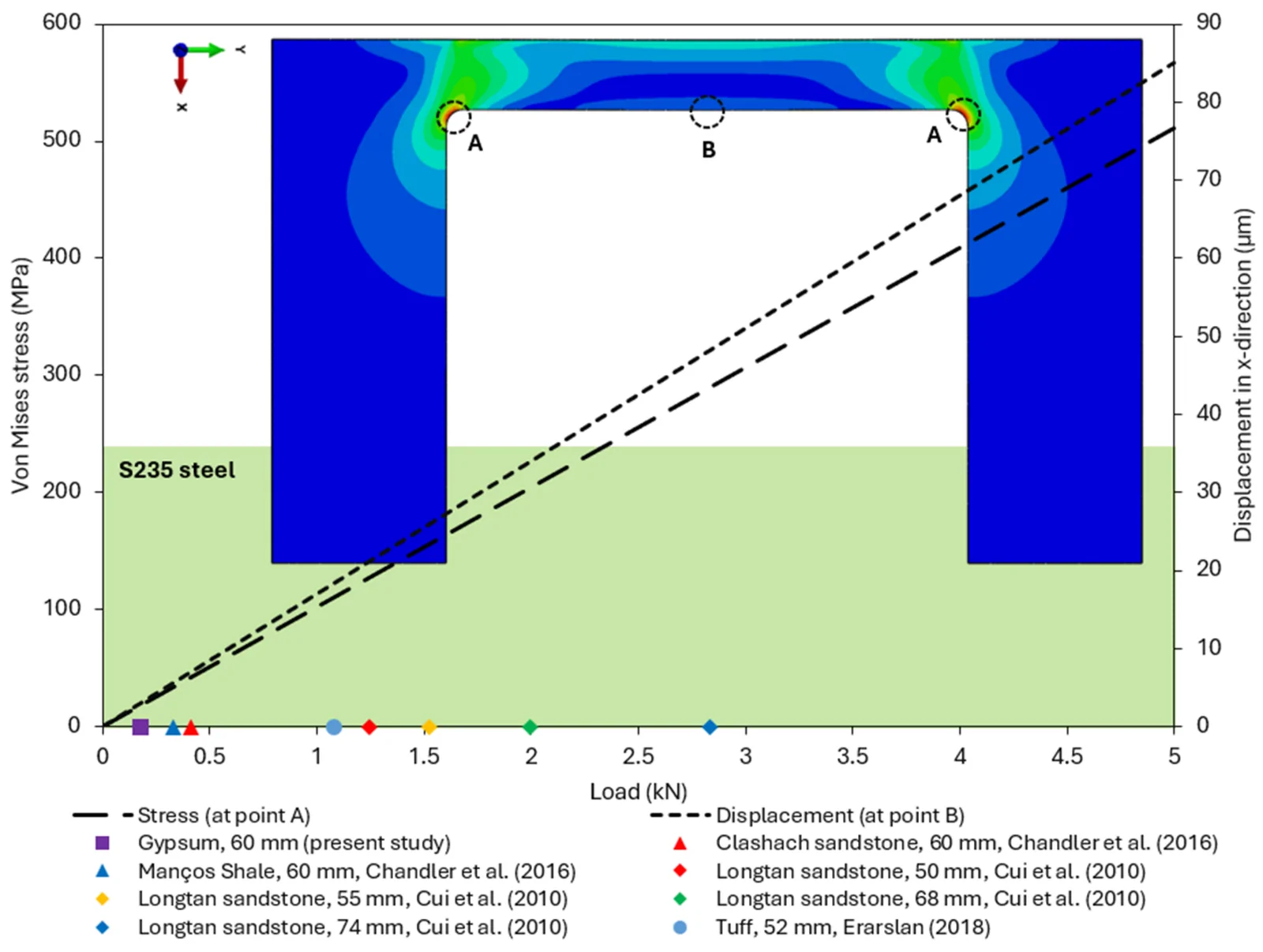
Von Mises stress and horizontal displacement of the U shaped plate under 5 kN load [26,40,41].

The FEM results show that, under an applied load of 5 kN, the highest stress concentration develops at the inner corners of the U-shaped plate. The maximum von Mises stress reaches approximately 511 MPa, while the corresponding horizontal displacement at the mid-span of the plate is about 85 µm. The middle plate is the second most highly stressed component, with a maximum stress of approximately 137 MPa. Figure 5 presents the von Mises stress distribution in the U-shaped plate, together with the corresponding load–stress and load–horizontal displacement responses. In practical applications, the fracture load of Short Rod specimens is generally below 5 kN. Cui et al. (2010) reported an average peak load of approximately 2835 N for Longtan sandstone specimens with a diameter of 74 mm, whereas 50 mm specimens failed at approximately 1246 N, indicating a clear specimen-size effect on the required fracture load [40]. Figure 5 also compares the fracture loads obtained in the present study with values reported for different rock types and specimen diameters [26,40,41]. The FEM results indicate that the S235 steel U-shaped plate remains below its yield strength at applied loads up to approximately 2.4 kN. This capacity is sufficient for the gypsum specimens tested in the present study. Accordingly, the present 5 mm S235 fixture is considered suitable primarily for specimens with relatively low fracture loads and should not be extended to stronger rocks requiring substantially higher loads without redesign. However, the suitability of the fixture should not be assessed solely based on elastic strength. Its stiffness and the amount of elastic strain energy stored during loading are also important, particularly when the post-peak response is of interest. Excessive deformation of the U-shaped plate may release stored energy rapidly after the peak load and destabilize crack propagation. This effect is less critical for Level I SR testing, where only the peak load is required for $K_{IC}$ determination. For Level II testing or other studies requiring reliable post-peak response capture, both the strength and stiffness of the fixture, particularly the U-shaped plate, should therefore be carefully considered. For specimens with higher fracture loads, the fixture should be redesigned using an appropriate combination of higher-strength material, increased plate thickness, and optimized geometry to limit both stress and elastic energy storage. The U-shaped and middle plates should also be resized for different specimen diameters. An excessive gap between the specimen and the U-shaped plate increases the bending action of the plate, resulting in greater deformation, higher energy storage, and increased stress concentration at the inner corners.

### 2.3. Short Rod Testing Using the ST Loading Fixture

**Figure 6:**
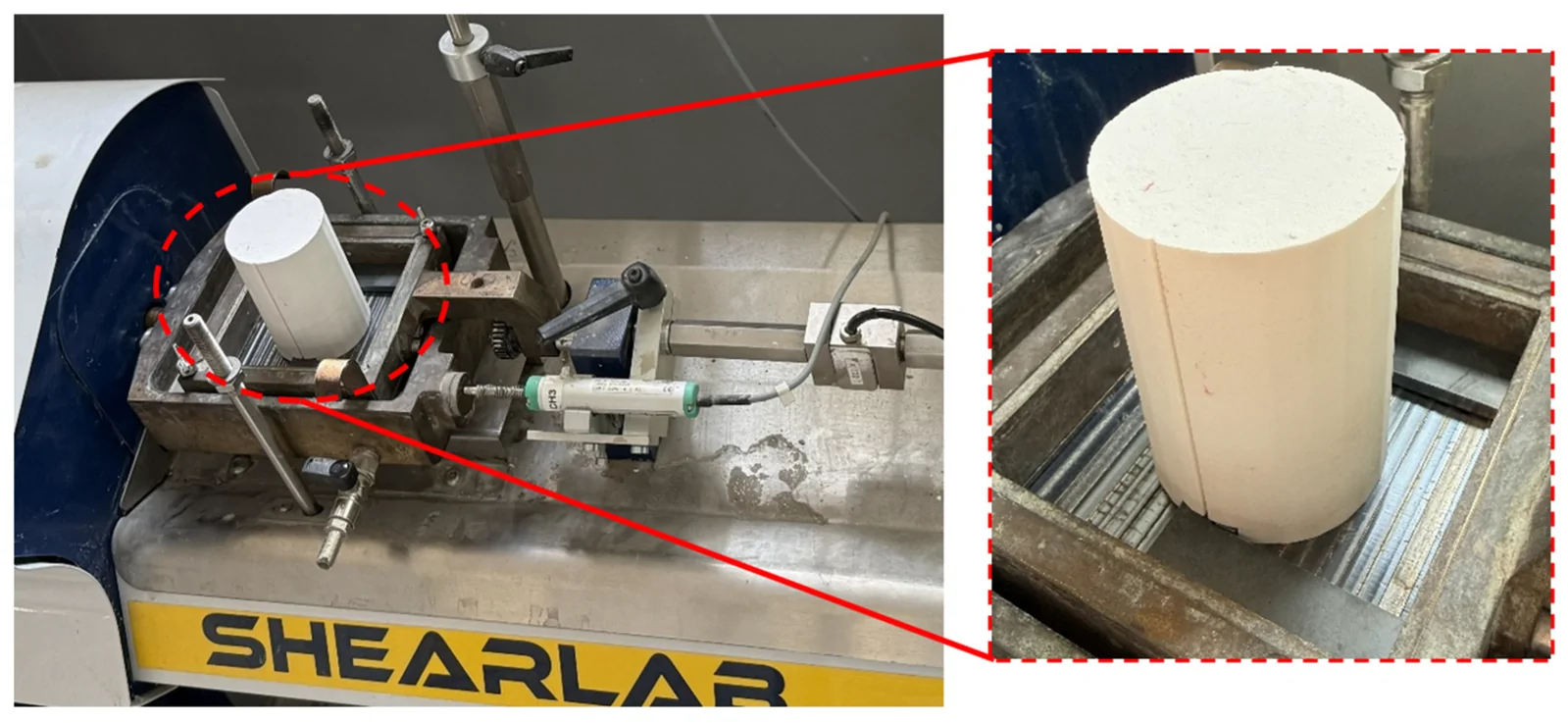
Short Rod test setup with the ST fixture installed in the direct shear apparatus.

The ST tool plates were first assembled inside the shear box, which was then installed in the direct shear apparatus. SR specimens were subsequently placed on the ST tool and properly seated before loading. Tests were conducted under displacement control at a constant rate of 0.1 mm/min to ensure quasi-static conditions [12,42,43]. Load and displacement were continuously recorded throughout the test, including the post peak stage. The complete test configuration is shown in Figure 6.

The peak load at failure was identified for each specimen and used to determine the Mode I fracture toughness, $K_{IC}$, following the ISRM recommended procedure for the Short Rod test [11,17]:

(1) $$K_{IC}=24.0\frac{P_{max}}{D^{1.5}}C_{K}$$

(2) $$C_{K}=1-0.6\frac{\Delta L}{D}+1.4\frac{\Delta a_{0}}{D}-0.01\Delta \theta$$

In these expressions, 24.0 represents the ISRM recommended dimensionless stress intensity coefficient for the standard SR geometry. $P_{max}$ denotes the recorded peak load, and D is the specimen diameter. The term $C_{K}$ is a geometric correction factor introduced to account for small deviations from the reference specimen dimensions. Here, $\Delta_{L}$ represents the variation in specimen length, $\Delta_{a0}$ is the deviation in the distance from the chevron notch tip to the specimen surface, and $\Delta_{\theta }$ denotes the deviation in the chevron notch angle. Although the tensile load is transferred through mechanical grooves rather than adhesively bonded end plates, the SR specimen geometry and nominal diametral tensile loading condition remain consistent with the ISRM configuration. Therefore, the standard dimensionless stress intensity coefficient of 24.0 was retained for the present calculations. For validation, fracture toughness values obtained from the SR tests were compared with those from SCB tests. The SCB method has been widely benchmarked against both direct and indirect approaches, generally showing good agreement. Compared with indirect methods such as the Edge-Notched Disc Bend (ENDB) and Chevron Cracked Notched Brazilian Disc (CCNBD) tests, SCB often yields lower $K_{IC}$ values, while giving slightly higher values than direct methods such as pCT [44,45,46,47].

### 2.4. DEM Simulation of Short Rod Testing

A three-dimensional discrete element model was developed using the open-source MUSEN framework [48] to investigate fracture development in the SR specimen under the proposed loading configuration. The specimen geometry, including the chevron notch, was reproduced according to the experimental dimensions and discretized using spherical particles. The Solid Elastic Bond model was used to represent the interaction between neighboring particles, allowing crack initiation and propagation to be simulated through progressive bond breakage. The model comprised approximately 124,000 particles and 1.42 million solid bonds. The micromechanical parameters were initially calibrated using UCS and BTS responses and then slightly refined against the experimental SR load–displacement response. The final parameters are summarized in Table 2.

**Figure 7:**
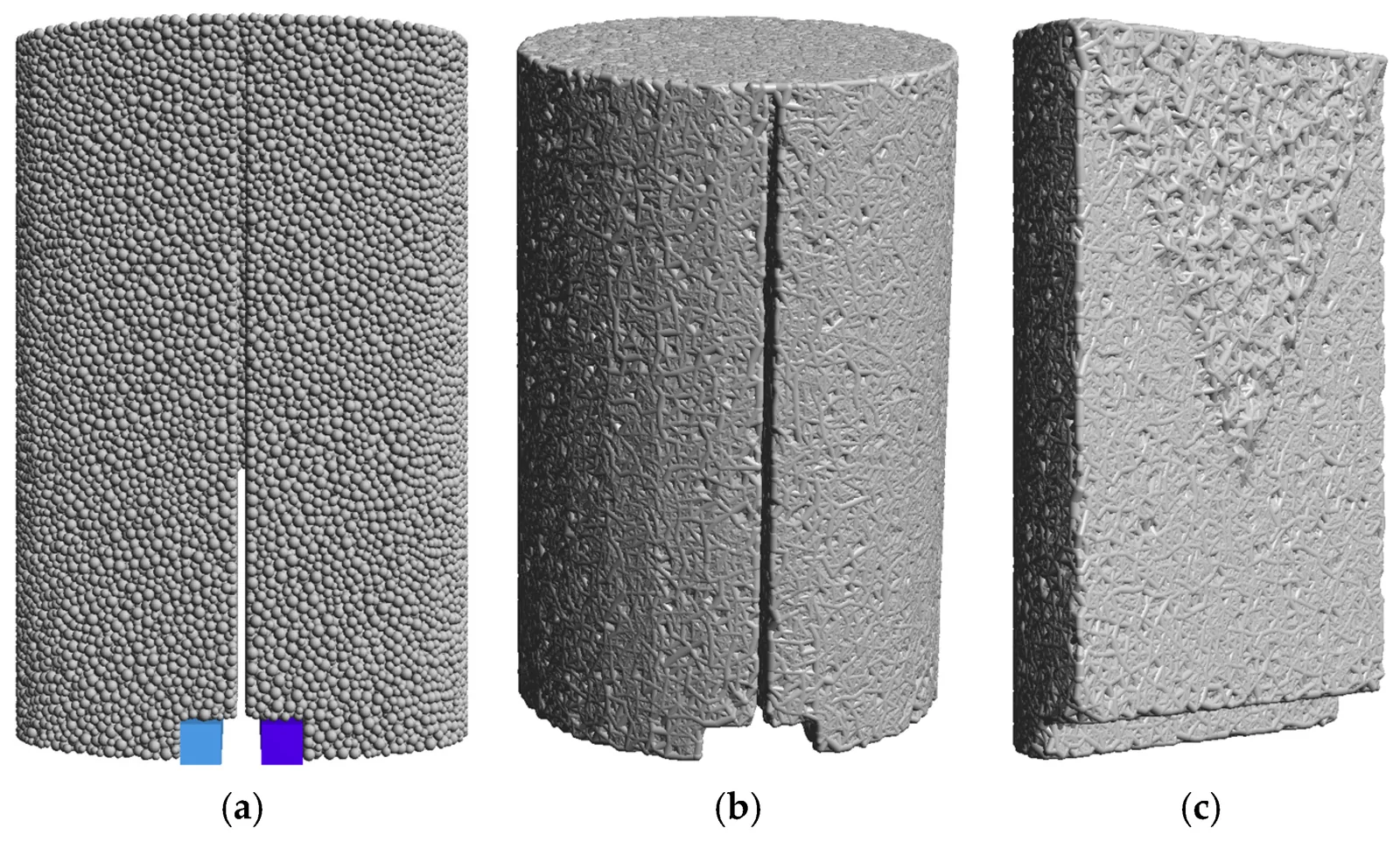
DEM model of the SR specimen: (**a**) particle assembly and loading plates, (**b**) bond network, and (**c**) cross-sectional view of the bond network.

Loading was applied through the specimen grooves following the experimental configuration. The DEM model is shown in Figure 7, while the load–displacement response and fracture evolution are presented in Section 3.

## 3. Results and Discussions

**Figure 8:**
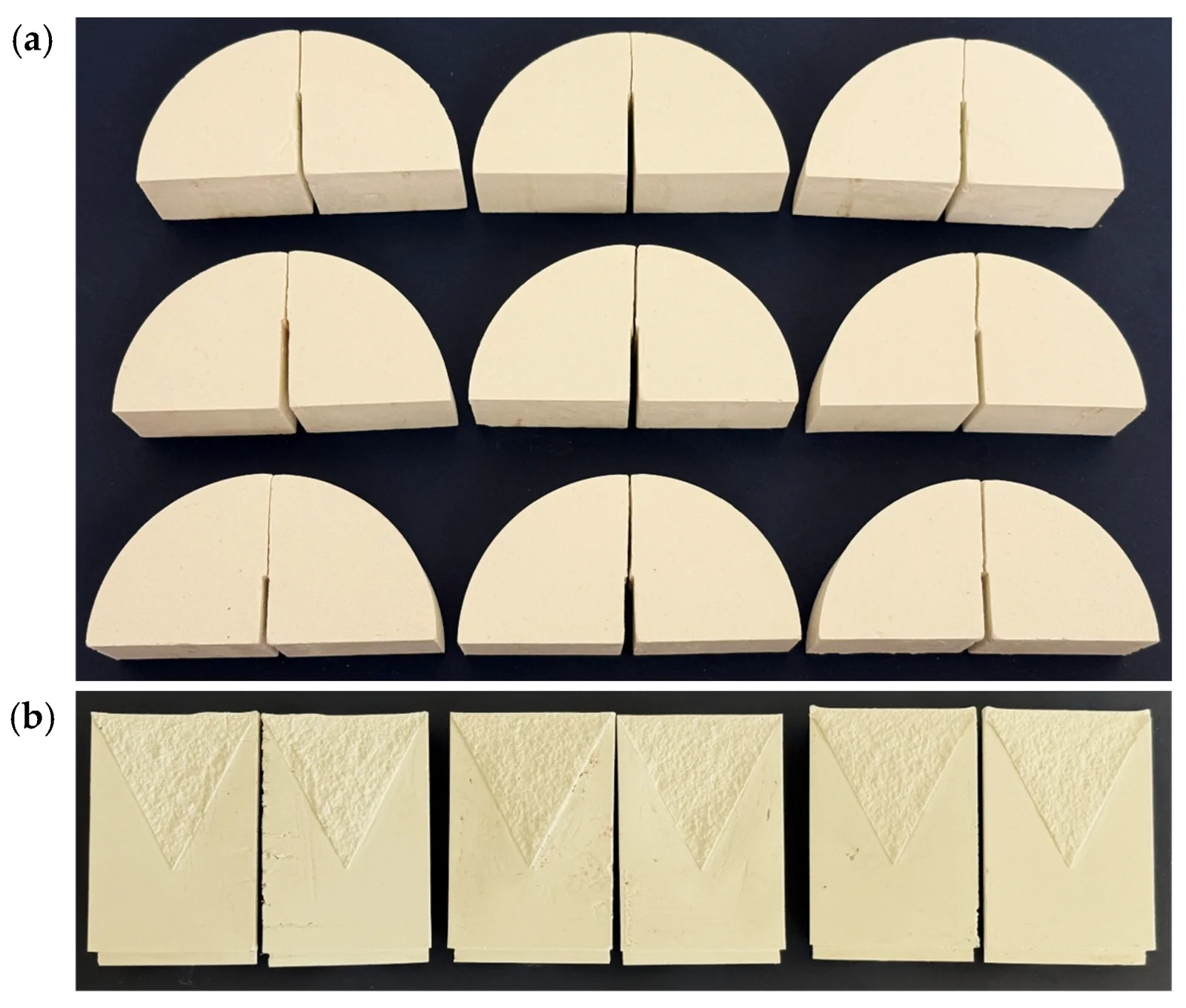
Observed fracture patterns in tested specimens: (**a**) SCB specimens and (**b**) SR specimens.

**Figure 9:**
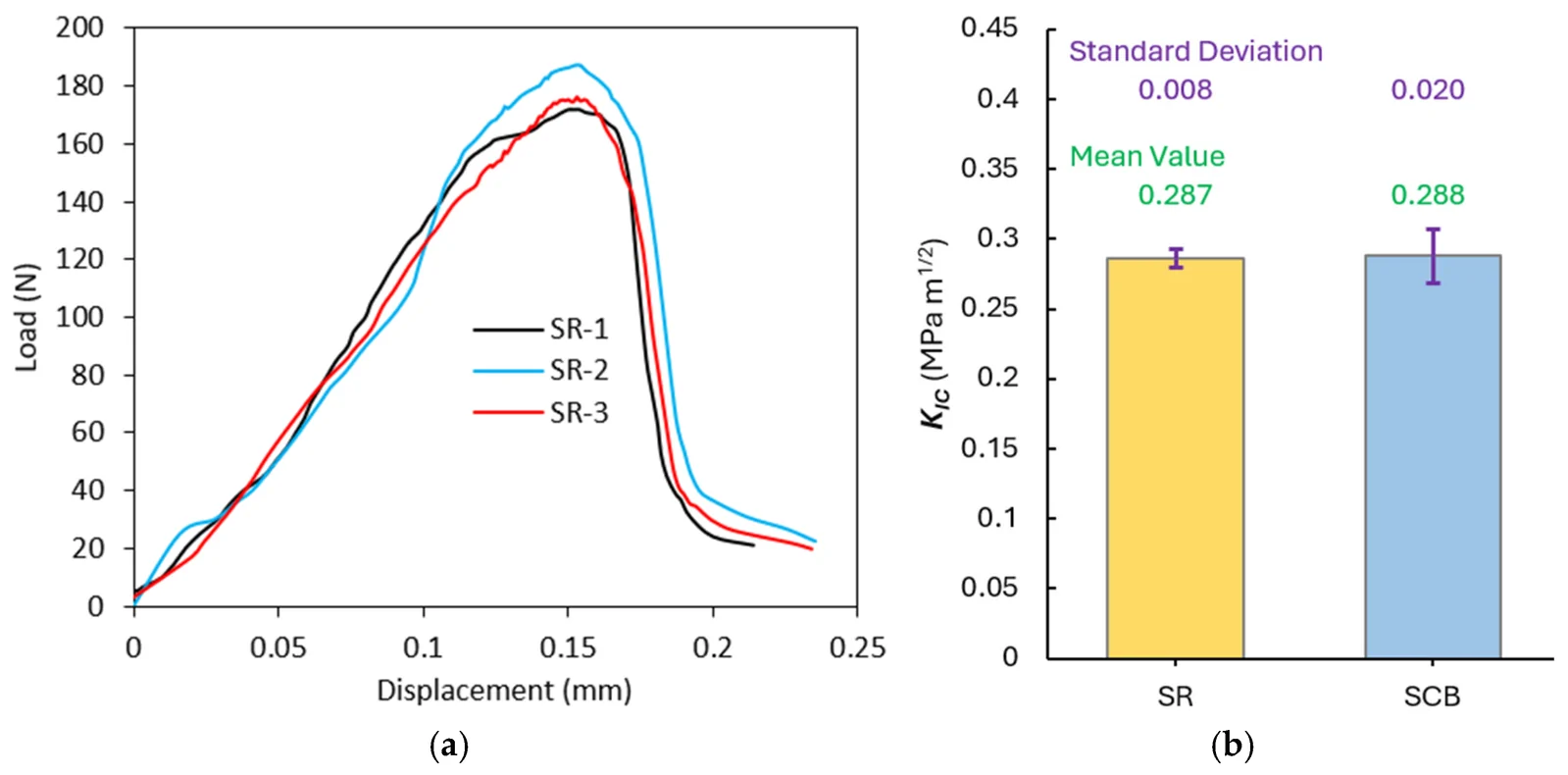
(**a**) Load–displacement curves obtained from SR tests. (**b**) Comparison of mean $K_{IC}$ values and standard deviations obtained from the SR and SCB tests.

Figure 8 illustrates the fracture patterns of the tested specimens. The SCB specimens (Figure 8a) failed into two nearly symmetric halves, with cracks initiating at the notch tip and propagating along the loading axis, confirming a predominantly Mode I tensile fracture. Similar behavior was observed in the SR specimens (Figure 8b), where cracks initiated at the chevron notch and propagated axially until complete separation, consistent with the expected Mode I fracture mechanism of the Short Rod test. Figure 9a presents the load–displacement responses of the SR specimens. The peak loads obtained from these curves were used to determine the Mode I fracture toughness, $K_{IC}$. The close agreement among the responses indicates good repeatability, which can be attributed to the consistent specimen geometry, homogeneous material, and stable load transfer provided by the ST fixture.

For the tested gypsum SR specimens, the fracture loads were well below the maximum load considered in the finite element analysis. At the representative fracture load of 181 N, the maximum von Mises stress at the inner corners of the U-shaped plate was approximately 25 MPa, far below the 235 MPa yield strength of S235 steel. The corresponding horizontal displacement was only 3.3 μm, indicating negligible fixture deformation. These results confirm that the ST fixture remained within the elastic range and that its deformation was unlikely to significantly affect the Level I response used for $K_{IC}$ determination. As shown in Figure 9b, the SR tests yielded a mean value of 0.287 MPa√m with a standard deviation of 0.008, while the SCB tests produced a mean value of 0.288 MPa√m with a standard deviation of 0.020. The excellent agreement between the two methods indicates that the proposed direct loading configuration provides fracture toughness values consistent with the established SCB method. This agreement can be attributed to the homogeneous microstructure and excellent geometric consistency of the cast gypsum specimens produced using 3D-printed molds. In addition, as a brittle material with relatively low tensile strength, gypsum is less sensitive to the additional stress components associated with the indirect loading conditions of the SCB test, resulting in nearly identical values.

**Figure 10:**
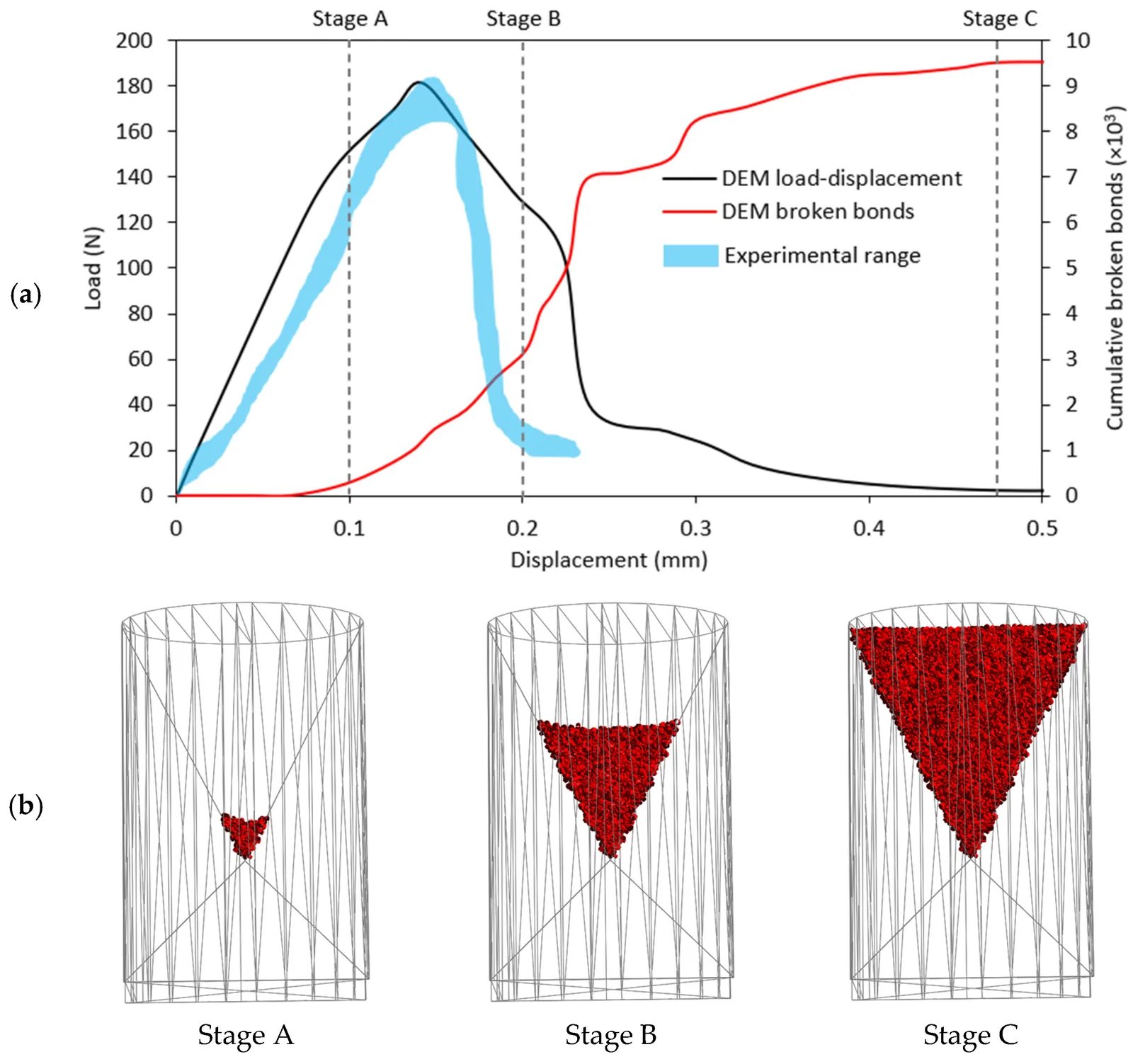
(**a**) DEM load–displacement response and cumulative broken bonds; (**b**) selected stages of fracture development in the SR specimen.

Figure 10 compares the DEM response with the experimental range and shows the evolution of cumulative broken bonds during fracture. The numerical model reproduced the experimental initial stiffness and peak load reasonably well. Bond breakage began near the chevron-notch tip before the peak load and increased as the crack propagated through the remaining ligament. Rapid bond failure accompanied the post-peak load drop, while the number of broken bonds gradually approached a plateau at larger displacements. The simulated fracture path was consistent with the experimentally observed SR fracture pattern.

The ST fixture converts the shear motion of the direct shear apparatus into tensile loading across the SR specimen. This concentrates stress at the chevron-notch tip, where fracture initiates and propagates through the remaining ligament. The DEM simulation captures the same progression, with bond breakage beginning before the peak load and increasing during post-peak crack growth. The simulated fracture path agrees with the experimental observations, supporting the intended Mode I fracture mechanism. The present study was limited to low-strength gypsum and should therefore be regarded as a proof of concept. Application to stronger rocks requires further experimental validation and appropriate fixture redesign for higher fracture loads.

## 4. Conclusions

In this study, a new loading system for Short Rod (SR) testing was developed and evaluated through experimental testing and numerical analyses. The system uses a shear-to-tensile fixture integrated with a conventional direct shear apparatus to apply direct tensile loading to SR specimens. The tests produced the expected Mode I fracture pattern and consistent load–displacement responses. The DEM analysis also captured progressive fracture development from the chevron-notch tip through the specimen ligament, in agreement with the experimental observations. The mean $K_{IC}$ obtained from the SR tests was 0.287 MPa√m, closely matching the 0.288 MPa√m obtained from SCB tests on the same gypsum. This agreement supports the feasibility of the proposed system for Level I SR testing. Since the validation was limited to specimens with relatively low fracture loads, the study should be regarded as a proof of concept. Extension to stronger rocks will require further experimental validation and fixture redesign for higher loading levels.

## Figures and Tables

**Table 1:** Geometrical parameters of SR and SCB specimens. D = diameter, B = SCB thickness, S = SCB support spacing, w = SR length, a_0_ = SR chevron tip distance from the loaded end, and a = SCB notch length. All dimensions are given in mm.

Test	n	*D*	*B*	*S*	*w*	*a*_0_	*a*
SR	3	60	-	-	87	29	-
SCB	9	75	30	50	-	-	15, 18, 21

**Table 2:** Main simulation and micromechanical parameters of the DEM model.

Parameter	Value	Parameter	Value
Particle diameter (mm)	1.0–1.6	Bond Young’s modulus (GPa)	1.5
Number of particles	123,756	Bond normal strength (MPa)	2.1
Number of solid bonds	1,417,080	Bond tangential strength (MPa)	6.3
Simulation time step (s)	$1\times 10^{-7}$	Particle/bond Poisson’s ratio	0.20
Particle density (kg/m^3^)	2000	Particle–particle sliding friction	0.45
Particle Young’s modulus (GPa)	5.0	Steel–particle sliding friction	0.45

## Data Availability

The original contributions presented in this study are included in the article. Further inquiries can be directed to the corresponding author.
